# The FapF Amyloid Secretion Transporter Possesses an Atypical Asymmetric Coiled Coil

**DOI:** 10.1016/j.jmb.2018.06.007

**Published:** 2018-10-12

**Authors:** Sarah L. Rouse, Fisentzos Stylianou, H.Y. Grace Wu, Jamie-Lee Berry, Lee Sewell, R. Marc L. Morgan, Andrea C. Sauerwein, Steve Matthews

**Affiliations:** Department of Life Sciences, Imperial College London, South Kensington Campus, London SW7 2AZ, UK

**Keywords:** functional amyloid transporter, *Pseudomonas*, Fap, coiled coil, NOESY, nuclear Overhauser effect enhancement spectroscopy, HSQC, heteronuclear single quantum coherence spectroscopy

## Abstract

Gram-negative bacteria possess specialized biogenesis machineries that facilitate the export of amyloid subunits, the fibers of which are key components of their biofilm matrix. The secretion of bacterial functional amyloid requires a specialized outer-membrane protein channel through which unfolded amyloid substrates are translocated. We previously reported the crystal structure of the membrane-spanning domain of the amyloid subunit transporter FapF from *Pseudomonas*. However, the structure of the periplasmic domain, which is essential for amyloid transport, is yet to be determined. Here, we present the crystal structure of the N-terminal periplasmic domain at 1.8-Å resolution. This domain forms a novel asymmetric trimeric coiled coil that possesses a single buried tyrosine residue as well as an extensive hydrogen-bonding network within a glutamine layer. This new structural insight allows us to understand this newly described functional amyloid secretion system in greater detail.

## Highlights

• We present the crystal structure of the FapF periplasmic domain, essential for function.
• NMR spectroscopy revealed an asymmetric conformation of a single Tyr in a trimeric coiled coil.
• The trimer was destabilized by mutation of a conserved R-h-x-x-h-E trimerization motif.
• Crystal structure reveals a functionally important glutamine hydrogen bonded network (Q-layer).

## Graphical abstract

**Figure:**
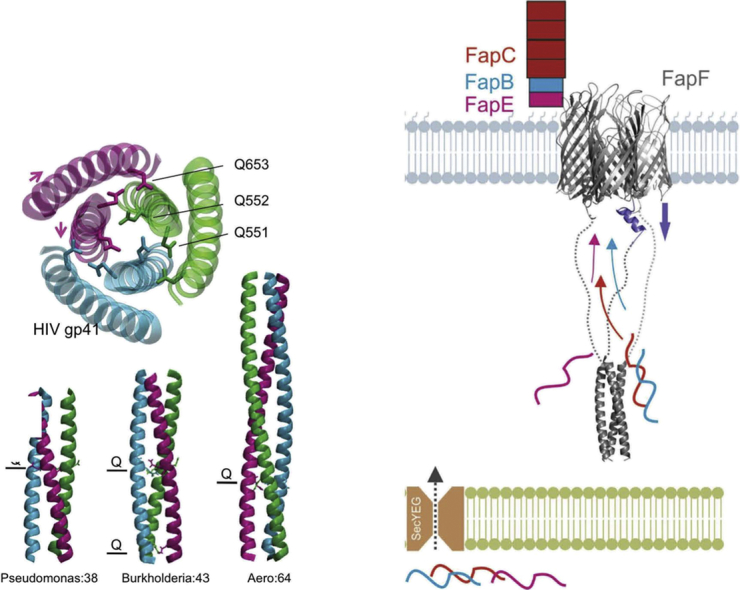

The formation and deposition of the amyloid state of proteins, amyloidogenesis, is a major cause of degenerative human disorders [1]. Microorganisms, especially bacteria, have exploited amyloidogenesis to drive the formation of functional protein assemblies which can be used to their advantage [2]. Bacteria use the unique structural properties of amyloid fibers to provide organizational integrity in and reinforce biofilms [3]. To utilize amyloid fibers for functional purposes without the cytotoxic effects, bacteria have evolved highly efficient pathways to transport amyloidogenic polypeptides across cells and membranes allowing for their assembly at the correct time and place. Understanding the mechanisms by which bacteria are able to control amyloidogenic proteins has important implications for human health and disease, both in terms of combating bacterial infections and by contributing to the wider understanding of amyloid formation and implications in diseases such as Alzheimer’s and Parkinson’s. Moreover, bacterial amyloid systems are emerging as a rich resource of inspiration for exploitation in biotechnology, most recently in the integration of amyloid secretion pores into DNA nanopore sequencing [4]

Functional amyloid has now been identified in many bacterial species [5]. Gram-negative bacteria possess particularly sophisticated amyloid secretion pathways, as they must shuttle amyloidogenic subunits across the periplasm and then through the outer membrane without the premature formation of toxic fibrils, intermediates or misfolded aggregates. The best characterized of these systems is that of curli [6], from *Escherichia coli*, for which three-dimensional structures of the majority of the components have been determined [7]. A genetically unrelated functional amyloid protein system (Fap) is found in many β-, δ-, and γ-proteobacteria [8] and in pathogenic strains and is thought to function as a virulence enhancing factor [9]. The molecular details of this more recently discovered system are only just beginning to be unpicked [10].

The Fap proteins are encoded by a single operon *fapABCDEF*. The major fiber-forming subunits are FapB and FapC, which are exported across the outer membrane *via* the membrane protein FapF, along with FapE which is also detected as a minor component associated with Fap fibers. FapA and FapD have putative roles in controlling the amyloidogenic properties of FapC and the processing of one or more Fap proteins, respectively, although their exact roles are yet to be determined [11].

We previously determined the crystal structure of the transmembrane domain of FapF, FapF_β_, through which the amyloid fiber subunits (FapC, FapB, and FapE) are exported. FapF_β_ forms a 12-stranded β-barrel, the aperture of which is plugged by an N-terminal helix. Full-length FapF possesses a large N-terminal periplasmic region comprising the first 70-amino-acid residues. A coiled coil is predicted in this region together with a ~ 40-amino-acid linker to the transmembrane domain. *In vivo* secretion assays have demonstrated that this periplasmic domain is essential for secretion by regulating the release of the helical plug [10]. Furthermore, the presence of this domain drives a shift from a predominantly monomeric species of FapF to a trimeric form. However, the precise roles of the coiled-coil domain in Fap assembly, substrate recruitment, and secretion are not yet clearly defined. We sought to understand the structural and mechanistic role of this coiled-coil domain by structural and biophysical techniques. Using a combination of X-ray crystallography and solution NMR spectroscopy together with molecular dynamics simulations, we show that the coiled coil forms a novel asymmetric trimer of parallel helices.

The oligomerization states of helical coiled coils are often influenced by solution conditions and the presence of additional residues or domains outside the coiled-coil region. The periplasmic domain of the FapF membrane transporter possesses a conserved *R-h-x-x-h-E* trimerization motif within predicted helical secondary structure between residues 3 and 40, herein referred to as FapF_CC_ [12] (Fig. 1a). To test the folding and multimerization properties of FapF_CC_, we expressed a series of recombinant peptides encompassing this region in *E. coli*, in addition to testing a synthetic peptide that encompasses only the coiled-coil region. CD, SECMALs (described in Ref. [8]) and NMR analysis of the synthetic and purified recombinant forms confirm a coiled-coil trimer with a melting temperature of 76.5 °C (Figs. 1b, e, S1). Disruption of the trimerization motif by either a R → A/E mutation was shown to disrupt the heterogeneity of the sample (Figs. 1D and S1) as well as reducing the *T*_m_ of the coiled coil to 64.5 °C and 53.5 °C for the R33A and R33E mutants, respectively (Fig. 1e). In contrast, replacement of the central tyrosine with alanine (Y20A) did not affect the secondary structure or stability of the coiled coil (Fig. S2). Similarly, extending FapF_CC_ with the FapF_CC_–FapF_β_, linker region did not introduce additional helical structure, suggesting that this region is disordered (Fig. S2) as predicted [10].

**Fig. 1:**
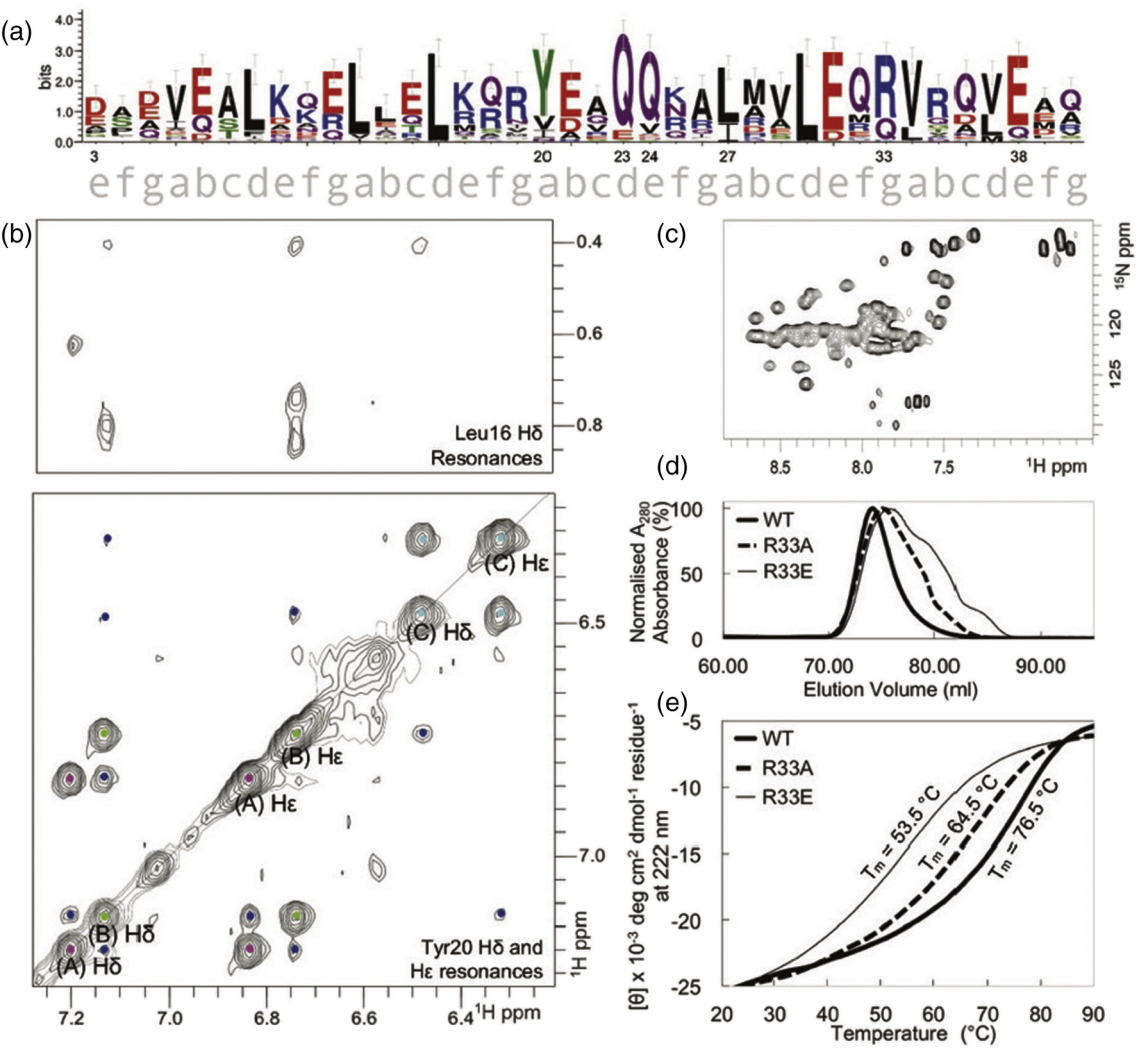
**(**a) The sequence of the coiled-coil region, residue 3–40, of FapF (FapF_CC_) used in this study. The coil heptad positions are shown below the plot. Sequence conservation was generated using weblogo [8], [9], [13]. (b) Aromatic regions from the ^1^H–^1^H NOESY NMR spectrum of FapF_CC_. Tyr20 diagonal peaks for each chain are labeled (a), (b), or (c) and dotted, colored according to their respective chain. Dots of the same color correspond to intra-Y20 NOE crosspeaks; inter-Y20 NOE crosspeaks are dotted in blue. (c) ^1^H–^15^N HSQC spectrum of ^15^N-labeled recombinant FapF_CC_. (d) Size exclusion profiles for recombinant FapF_CC_ wild type, R33A and R33E. (e) CD melting curve profiles for FapF_CC_ wild type, R33A and R33E.

We next set out to solve the crystal structure of FapF_CC_. Commercial sparse matrix screens identified crystals in two zinc acetate containing conditions which were subsequently optimized. Crystals grew rapidly to up to 200 μm^3^ over the course of 3 days and diffraction data were collected to 1.8 Å. Data were initially autoindexed by the Xia2 3dii pipeline [45], in space group *H*32, and a solution for residues 5–38 was found using MoRDa [14], but refinement failed to progress with R values remaining high (*R*_free_ > 30%). To investigate this further and gauge the conformational heterogeneity of FapF_CC_, we recorded a 2D ^1^H–^1^H nuclear Overhauser effect enhancement spectroscopy (NOESY) NMR spectrum (Fig. 1b). To our surprise, although the FapF_CC_ contains only a single aromatic residue, Tyr20, three resolved tyrosine spin systems could be identified. This indicated that the structure of FapF_CC_ is asymmetric. We therefore subsequently processed the diffraction data in P1 to model the six chains in the asymmetric unit individually (Fig. S3) and the structure could be refined using REFMAC5 [15] and PHENIX [16] with a final *R*_free_ of 24%. Full-data collection and refinement statistics are shown in Table 1

**Table 1:** Data collection, phasing and refinement statistics

	FapF_CC_
**Data collection**	
Space group	*P*1
Cell dimensions	
*a*, *b*, *c* (Å)	37.59, 37.70, 57.34
*α*, *β*, *γ* (°)	90.24, 108.95, 119.89
Wavelength	0.97625
Resolution (Å)	1.78
*R*_pim_	0.027 (0.282)
*I*/σ*I*	13.86 (2.4)
Completeness (%)	93.5
Redundancy	2.0

**Refinement**	
Resolution (Å)	1.78
No. reflections	22,547 (2213)
*R*_work_/*R*_free_	0.2145/0.2437
No. atoms	2140
Protein	1846
Ligand	21
Ramachandran favored (%)/allowed/outliers	99/0/9/0
Protein chain beta factor	37.27
Ligand	66.74
Water	47.21
R.m.s. deviations	
Bond lengths (Å)	0.02
Bond angles (°)	1.86

Data from a single crystal were used to solve the structure.

Values in parentheses are for highest-resolution shell.

The overall architecture of the trimeric, parallel coiled-coil assembly is shown in Fig. 2a. As the ^1^H–^1^H NOESY spectra clearly show three tyrosine residues in distinct conformations that are not exchanging on the NMR timescale (Fig. 1b), we were able to confidently model the core of the coiled coil with a single buried Tyr20 side chain per trimer (Fig. 2b). The final geometry of the three tyrosines within the crystal structure is also consistent with the NOE correlations between upfield shifted methyl groups of the Leu16 and Tyr20 side-chains protons (Fig. 1b). This leads to an asymmetric arrangement of two surface-exposed tyrosine residues within the trimer and the configuration allows the buried Tyr20 side chain to coordinate with a Gln23 side chain of an adjacent chain (Fig. S4A). To characterize this further, molecular simulations of the trimeric coil in 0.15 M NaCl solution confirm that the asymmetric tyrosine arrangement remained stable for the duration of three independent 100 ns simulation (Fig. S4B). Furthermore, control simulations in which all three Tyr20 residues were modeled in the outward facing conformation led to rearrangement of a single Tyr20 into the buried conformation, with the transition beginning within 5 ns and completing by 20 ns (Fig. S4C). The reverse transition from a buried Tyr20 to interfacial was not observed in any of the simulations, highlighting that the single buried Tyr20 represents a stable form of the trimer.

**Fig. 2:**
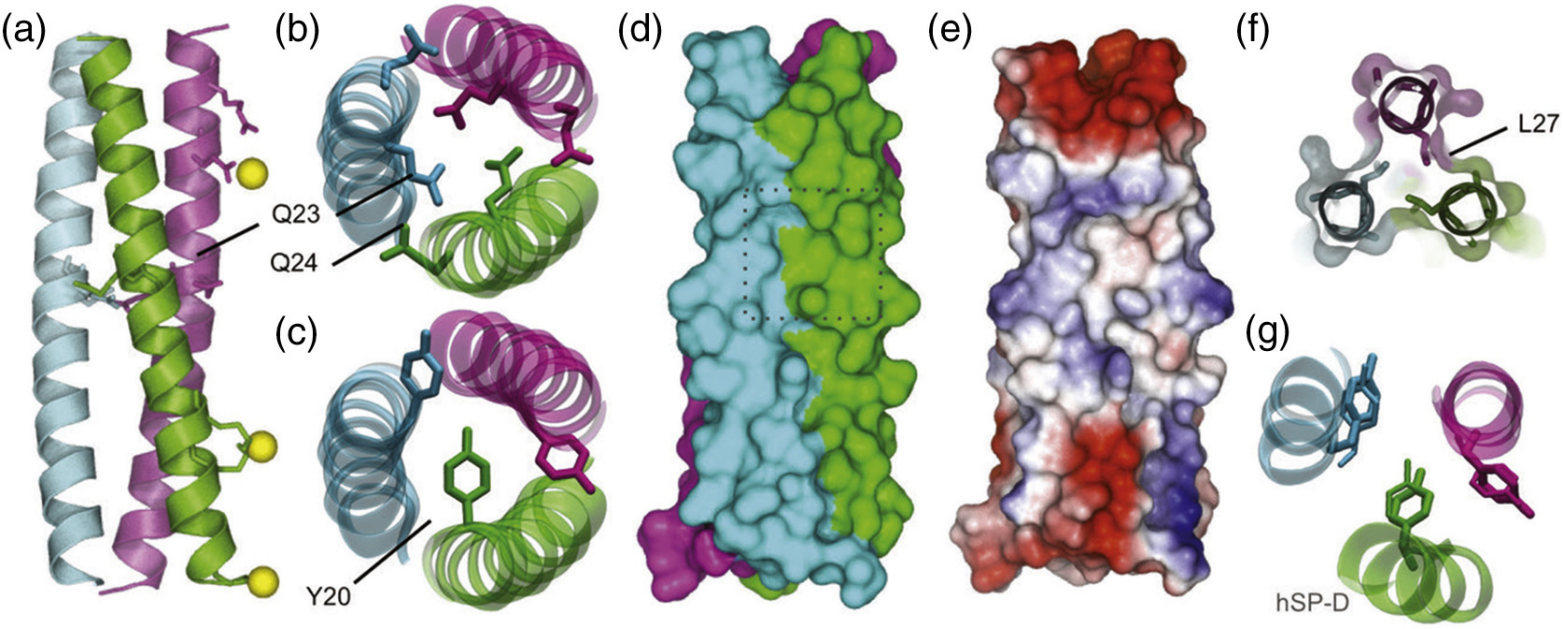
FapF_CC_ forms an asymmetric trimeric coiled coil. (a) Overall structure of the periplasmic domain of FapF, FapF_CC_. FapF_CC_ forms a parallel trimeric coiled coil. Coiled-coil charged surface residues interacting with Zn^2 +^ (yellow spheres) are highlighted for a single chain. Zinc coordination was validated using the checkmymetal server [17] and consistent with molecular simulation data (Fig. S3). (b) Buried Gln23 residues form a hydrogen bond network with interfacial Gln24 of neighboring chains (Q-layer, shown in more detail in Fig. S5). (c) FapF_CC_ contains a single buried Tyr20. (d) The surface of the periplasmic domain highlighting a large hydrophobic cleft in the center of the coil. (e) Surface view colored according to charge to further highlight the hydrophobic cleft. (f) Cutaway view of the hydrophobic cleft indicated in panel D showing the exposed Leu27 side chains. (g) Overlay of the asymmetric tyrosine arrangement of FapF_CC_ (thin lines) with human lung surfactant protein D (PDB ID 1b08 [18]) (thick lines and cartoon). Figures generated using Pymol [19] and VMD [20].

Despite the asymmetry of the central tyrosine residues, the conformation of the backbone for the three peptide chains is symmetric, as the number of backbone amides peaks observed in the ^1^H–^15^N heteronuclear single quantum coherence spectroscopy (HSQC) NMR spectrum is consistent that expected for single peptide chain (Fig. 1c). Such localized asymmetry in parallel coiled coils is rare, and in a search of the Coiled Coils Database [21], we found only a single previous example of a coiled-coil structure with a single buried Tyr in the human lung surfactant protein D [18] (Fig. 2d). However, in solution NMR, identical tyrosine chemical shifts were observed for all chains, suggesting that the asymmetric conformation was a consequence of crystallization. Despite this, the authors concluded the asymmetry, which propagates to the terminal lectin domains, could represent a functionally active state. In FapF_CC_, such asymmetry is observed in both solution and in crystalline conditions and therefore must be considered an intrinsic property of the coiled coil, which supports the suggestion of a functionally important role. By analogy with human lung surfactant protein D, the asymmetry of the tyrosine residues in FapF_CC_ may induce asymmetry in the C-terminal transmembrane domain, likely allowing the plug domains to open independently. Furthermore, asymmetry in the surface properties of FapF_CC_ (Fig. 2e) could enable the recruitment of other Fap proteins in a sub-stoichiometric ratio. It is worth noting that much of the surface is polar as expected; however, the central portion of the protein displays a surface hydrophobic pocket (Fig. 2f) that could represent a key binding site.

Another striking feature of FapF_CC_ is the presence of the buried polar Gln23 residues, which are stabilized by neighboring Gln24 residues through an extended hydrogen bonded network across the core of the protein (Fig. 2b). A central density between the Gln23 side chains in this “glutamine layer” (Q-layer) could be modeled as a water molecule. To explore the dynamics of this central region further, atomic resolution molecular simulations of the coiled coil were used to assess stability and dynamics (Fig. S5). The central water molecule was released upon simulation in solution conditions and likely corresponds to a trapped molecule under the cryogenic crystal conditions. Gln23 and Gln24 together are coordinated sufficiently, forming an extended hydrogen bonding network across the core of the coiled coil to form the Q-layer. Furthermore, analysis of the Gln23–Gln24 side-chain dynamics indicates that side-chain flipping occurs on the ns timescale (Fig. S5). The hydrogen bonded network is extended to include the Tyr20 residues (Fig. S5).

Although buried polar residues are underrepresented in coiled coils, glutamine and asparagine layers have previously been identified in coiled coils and have been linked to function. A notable example is the fusogenic core of the protein envelope of retroviruses [22]. Specifically, fusion-active gp41 from HIV-1 adopts a trimer of helical hairpins (Fig. 3a), in which three central N-terminal helices form a trimeric coiled coil surrounded by three antiparallel C-terminal helices [23]. The hydrogen bond network across this Q-layer modulates the stability of the hairpin arrangement and its disruption affects membrane fusion activity. A similar role for the ionic layers of SNARE helical bundles has been proposed in the recycling of intracellular vesicle fusion complexes [28]. Coiled-coil regions with polar hydrogen-bonded layers have been shown to exhibit enhanced dynamics [29], and this concept has been proposed for glutamine layers in nucleoporin coiled-coil complexes within the nuclear pore, in which they lower free-energy barriers for alternative interactions and packing of helices [30].

**Fig. 3:**
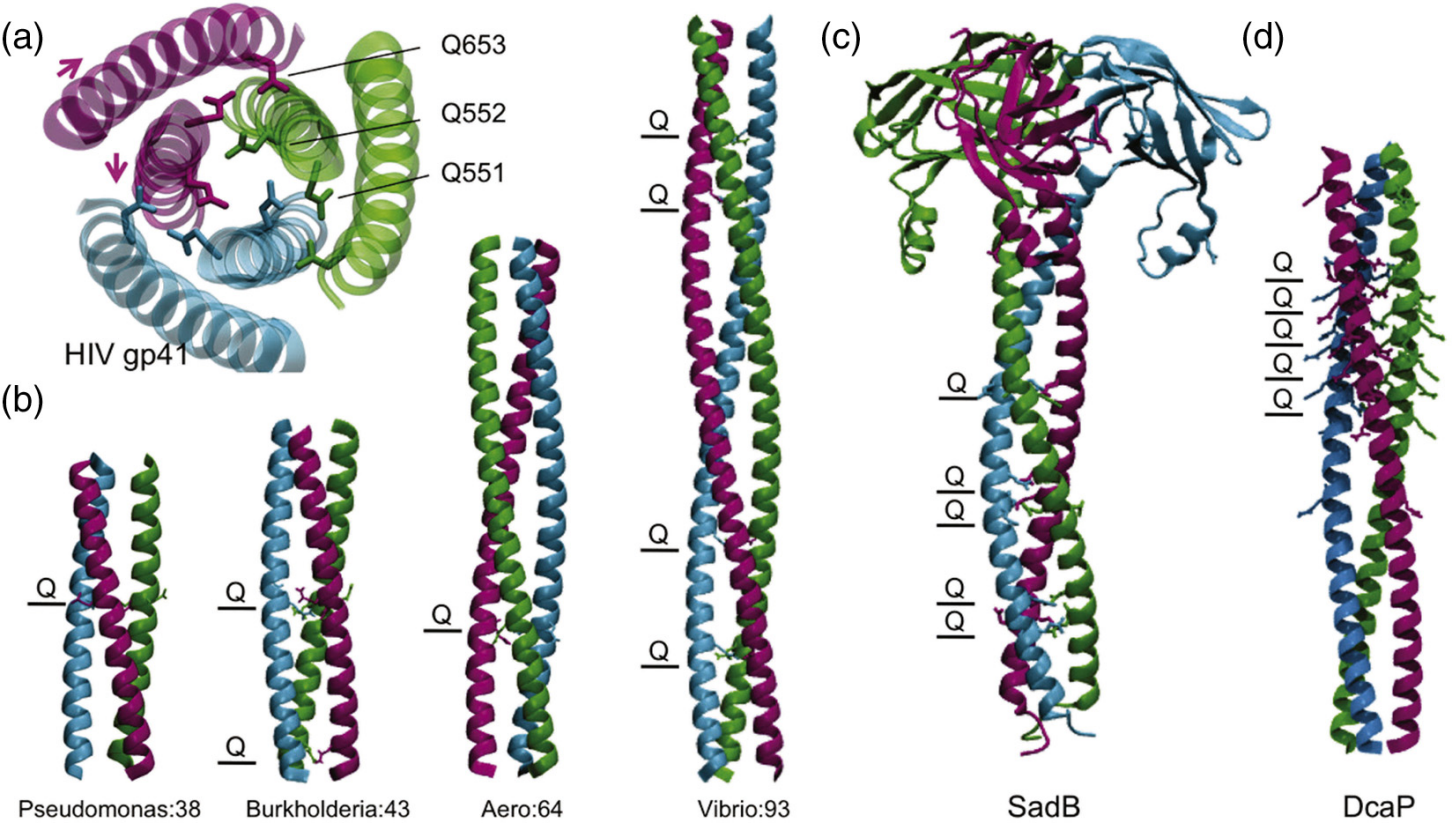
Q-layers in coiled coils. (a) The HIV gp41 (PDB id 1aik [23]) Q-layer extends to mediate packing interactions with other helices. The opposing direction of helices is indicated by arrows from N- to C-termini. (b) Periplasmic coiled-coil domains of FapF homologues using alignment data [8], [9], [13]. Coiled-coil prediction used LOGICOIL [24]. Models were built using CCBuilder v2.0 [25]. Buried polar Gln residues are highlighted. The number of expected residues in each case is shown. (c) The SadA autotransporter-associated protein SadB (PDB 4c47 [26]) has multiple double Q-layers. (d) Model of DcaP periplasmic domain built as for panel A [27].

The trimeric autotransporter adhesins of gram-negative bacteria also possess polar residues (usually asparagine) predicted to form buried layers in the coiled-coil domains. It has been suggested that these features modulate the folding, stability and dynamics of the coiled-coil domain which in turn maintains the passenger domain in a soluble and export-competent state [31]. After export, stabilization of these polar coiled-coil regions could then be triggered by final folding of the extracellular trimeric autotransporter adhesin. Intriguingly, a novel coiled-coil lipoprotein, SadB, shown to assist in the passage of the large, trimeric autotransporter SadA also possesses prominent Q-layers (Fig. 3c) [26]. No specific role for the Q-layers was ascribed, but the dynamic nature of coiled-coil regions possessing buried polar residues may promote transient interactions with the passenger domains during transport across the periplasm. The multimeric assembly of Q/N-rich prions and expanded polyQ sequences en route to fibrils and pathogenesis has also been shown to involve dynamic intermediate helical structures, presumably via the formation of extensive Q/N hydrogen bond networks [32].

An important question in the mechanism of Fap secretion is why FapF exists as a trimer and if all pores are active simultaneously. An asymmetric arrangement of the periplasmic coiled coil suggests that the plugs of the trimer FapF transmembrane barrel could be opened and closed independently. Furthermore, the potential destabilizing effect of conserved Q-layers in FapF coiled coils could promote the exchange between alternative conformations and translate to gating the transmembrane barrel plug. It has been shown for the sodium channels that opening occurs by the zipping of adjacent coiled-coil regions [33]. All FapF homologues possess coiled-coil domains with Q-layers. There is also a wide variation in the length of the predicted coiled coil and the number of Q-layers from other species (Fig. 3b), and this is often correlated with the abundance of Q/N-rich repeats within the principal amyloid substrate of the Fap systems. The outermembrane porins DcaP and ScrY are stabilized by glutamine-rich, trimeric coiled coils within the periplasm [27], [34] (Fig. 3d). Although direct roles in substrate translocation are unclear, it has been suggested that the coiled-coil domains act as molecular guides to an innermembrane component. Together with the similarity with SadB and its role in assisting autotransport of SadA, it is tempting to speculate that the coiled coil of the Fap channel plays a similar role in guiding Fap amyloid substrate transport from deep within the periplasm to the cell surface (Fig. 4).

**Fig. 4:**
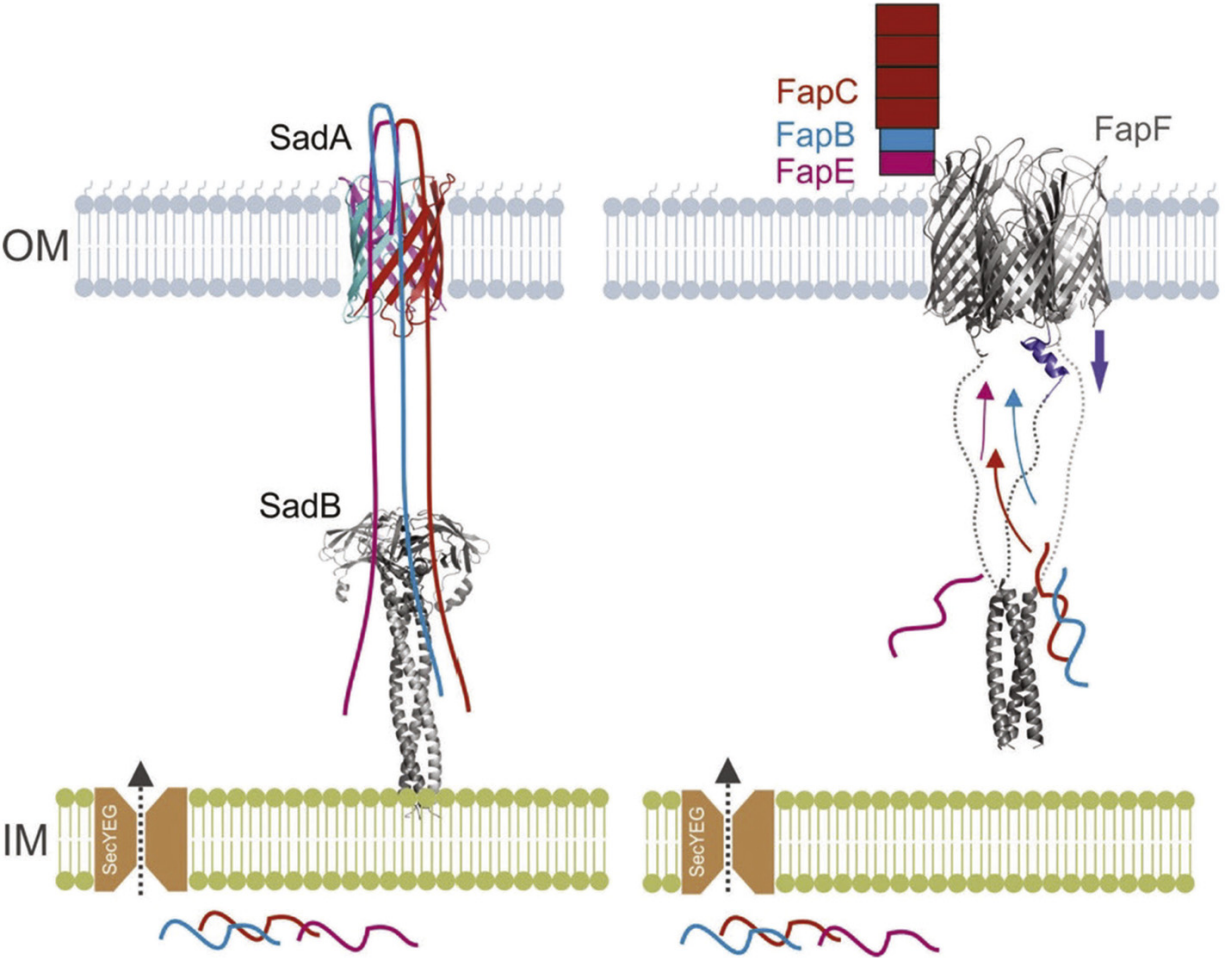
Schematic representation comparing the role of the coiled-coil domains and FapF domain and SadB. The coiled-coil domain of SadB has been suggested to play a role in maintaining the autotransporter SadA in a secretion competent from (*left*[26]). The coiled-coil domain FapF could play a similar role in Fap substrate guidance and secretion (*right*[10])

## Material and Methods

### Crystal preparation, data collection and structure determination

The synthetic peptide corresponding to the predicted coiled-coil region of FapF was synthesized at 97.5% purity DVDIETLKQELLELKQRYEAQQKALAVLEQRVRQVEDQ (ChinaPeptides). The peptide was resuspended to a final concentration of 10 mg ml^− 1^ (Nanodrop) and tested in a range of sparse crystallization screens as described elsewhere using Mosquito [35]. Crystals were only observed to form in two zinc acetate containing conditions over 3–5 days of which only one was of suitable diffraction quality [JSCG + 0.2 M zinc acetate dihydrate, 0.1 M sodium cacodylate (pH 6.5), 10% v/v 2-propanol]. Secondary screens across pH range 6.2–6.8 and zinc acetate concentration 0.1–0.3 M gradients led to crystals of 200 μM across. Crystals were harvested and flash frozen using superloops (MiTeGen). Data was collected from a single crystal on the Diamond i03 beamline. Crystals diffracted to 1.8-Å resolution. Data collection statistics are shown in Table 1. Data were automatically processed using the Xia2 pipeline into the *H*32 space group. Attempts to solve the structure by molecular replacement using existing trimeric parallel coiled-coil models found using the CC + database as search models were unsuccessful. This included our previously described model of FapF coil generated using CCBuilder v.1 [25] despite the final structure being similar to the final solved structure (Fig. S5). The AMPLE and MORDA pipelines [14] on CCP4 gave partial solutions with an eventual solution of all residues D3–E38 being found by MORDA. The data were reprocessed into *P*1 space group to allow for individual modeling of each of the six chains in the asymmetric unit. The data did not show significant twinning and could not be processed from *H*32 into *C*2 as reported elsewhere for other coiled coils [36]. Refinement was carried out using Refmac5 [15] and Phenix [16]. One hundred percent of residues were in allowed Ramachandran regions. One trimer (chains ABC) was fully resolved, whereas for the second (chains DEF), the termini could not be fully resolved. Chains ABC were therefore used for subsequent analysis.

### Molecular simulations

Atomistic simulations were run using the Gromacs 4.6.7 package [37] (www.gromacs.org) with the GROMOS53a6 force field [38]. The X-ray structure (chains ABC) was used as the starting model, and crystallographic water molecules, Na^+^ and Zn^2 +^ were retained. The system was solvated using the SPC water model, and ions were added to give a neutral system with a final NaCl concentration of 0.15 M with an initial box size of 8 × 8 × 8 nm. Periodic boundary conditions were applied, with a simulation time step of 2 fs. Equilibration runs were performed of 1 ns with a time step of 2 fs. The protein backbone was restrained, pressure was coupled at 1 bar using the Berendsen barostat [39], and temperature was maintained at 310  K using a V-rescale thermostat [40] with a coupling constant of 0.1  ps. For the 100-ns production runs, the pressure was controlled at 1  bar through coupling to a Parrinello–Rahman barostat [41] with a coupling constant of 1 ps. Particle Mesh Ewald was used for long-range electrostatics [42], and the LINCS algorithm was used to constrain covalent bond length [43].

### Cloning, expression and purification of recombinant protein

*Pseudomonas* UK4 FapF3–40 and mutants (R33A, R33E) were cloned into a pNIC28-Bsa4 vector (Addgene plasmid no. 26103) [44] with a N-terminal His-tag and TEV cleavage site, sequence MHHHHHHSSGVDLGTENLYFQ*SM (* = TEV cleavage site). BL21 (DE3) cells transformed with FapF_CC_ and mutants were grown in LB medium at 37 °C overnight. The culture was diluted 1:50 into 1 L of unlabeled LB or labeled M9 minimal medium [48 mM Na_2_HPO_4_, 22 mM KH_2_PO_4_, 8.6 mM NaCl (pH 7.4), 0.1% (w/v) ^15^NH_4_Cl, 1 mM MgSO_4_, 0.1 mM CaCl_2_, 0.2% (w/v) glucose] and grown at 37 °C 200 rpm. At OD_600_ = 0.8, protein overexpression was induced with 0.75 mM IPTG and left for 12–16 h at 30 °C 200 rpm. Cells were harvested and resuspended in lysis buffer [20 mM Tris–HCl, 300 mM NaCl (pH 8)] for lysis by sonication. The supernatant was separated by centrifugation at 16,000*g* for 30 min and purified by nickel-affinity chromatography, eluting with lysis buffer containing 250 mM imidazole. The His6-tag was removed by TEV protease cleavage and cleaved FapF3–40 purified by nickel chromatography, followed by gel filtration using a Superdex-75 column (GE Healthcare). FapF_CC_ samples for biophysical analysis were exchanged into phosphate buffer [20 mM PO_4_, 150 mM NaCl (pH 6.8)], and for ^1^H–^1^H–based NOESY experiments, the sample was exchanged into 100% D_2_O-based phosphate buffer using a Superdex 75 10/300 GL column (GE Healthcare) and several filtration runs (3-kDa molecular weight cutoff). Samples were concentrated for biophysical analysis.

### NMR spectroscopy

The FapF_CC_ peptide was prepared for the ^1^H–^1^H NOESY experiment (130 ms mixing time; 40 scans) by dissolving 1 mg into 400 μL 100% D_2_O-based phosphate buffer. ^1^H–^15^N HSQC experiments for recombinant ^15^N-labeled FapF_CC_ were carried out in 20 mM Tris–HCl, 300 mM NaCl and 10% D_2_O buffer (pH 8) with a sample concentration of 100 μM and 348 scans. The ^1^H–^1^H NOESY and ^1^H-^15^N HSQC experiments were recorded at 293 K, using the Bruker Avance III HD 800- and 600-MHz spectrometers equipped with triple-resonance cryoprobes, respectively. Spectra were processed within TopSpin 3.2 (Bruker) and analyzed using CcpNmr Analysis [46].

### Circular dichroism

Non-denaturing CD recordings for the peptide and recombinant FapF3–40 were carried out at 45 μM in phosphate buffer, as previously described [10]. Denaturation was monitored by CD ellicipity at wavelengths of 200 to 260 nm, with intervals of 0.5- and 1 nm bandwidth. The scan length per point was 5 s and three repeats were collected for each sample The temperature was increased by 1 °C per minute with a tolerance of 0.2 °C starting at 20 °C and ranging to 90 °C. For *T*_m_ determination, the maximum of the first derivative of the melting curve at 222 nm was taken.

### SEC-MALS

Size Exclusion Chromatography Multiple Anomalous Light Scattering (SEC-MALS) was performed using a Superdex 75 10/300 GL column (GE Healthcare), MiniDawn TREOS (Wyatt Technology) and OptiLab T-rEX (Wyatt Technology). The column was equilibrated with phosphate buffer and 100 μl FapF_3–40_ samples at concentration 1 mg/ml (in phosphate buffer) injected into the column with a flow rate of 0.25 ml min^− 1^. The refractive index increment (dn/dc) was set to 0.185 mL/g, and the weight-average molar masses determined with ASTRA software (Wyatt Technology).

### Accession Numbers

The structure was deposited in the PDB with accession number 6FUE.

## Appendix A Supplementary data

Supplementary figures Image 1
