# Supplementary material for: The FapF Amyloid Secretion Transporter Possesses an Atypical Asymmetric Coiled Coil
Source: J Mol Biol. 2018 Oct 12;430(20):3863–71. doi: 10.1016/j.jmb.2018.06.007 (PMC6173795; doi:10.1016/j.jmb.2018.06.007)
Supplement: Supplementary file 1 — Supplementary figures [file mmc1.docx]

**Supporting Information for:**

**The FapF amyloid secretion transporter possesses an atypical asymmetric coiled coil**

Sarah L. Rouse, Fisentzos Stylianou, Grace Wu, Jamie-Lee Berry, Lee Sewell, R. Marc L. Morgan, Andrea C. Sauerwein, Steve Matthews

Department of Life Sciences, Imperial College London, South Kensington Campus, SW7 2AZ

**Correspondence to Steve Matthews:** Department of Life Sciences, Imperial College London, South Kensington Campus, SW7 2AZ; email [s.j.matthews@imperial.ac.uk](mailto:s.j.mathews@imperial.ac.uk)

**Figure S1**


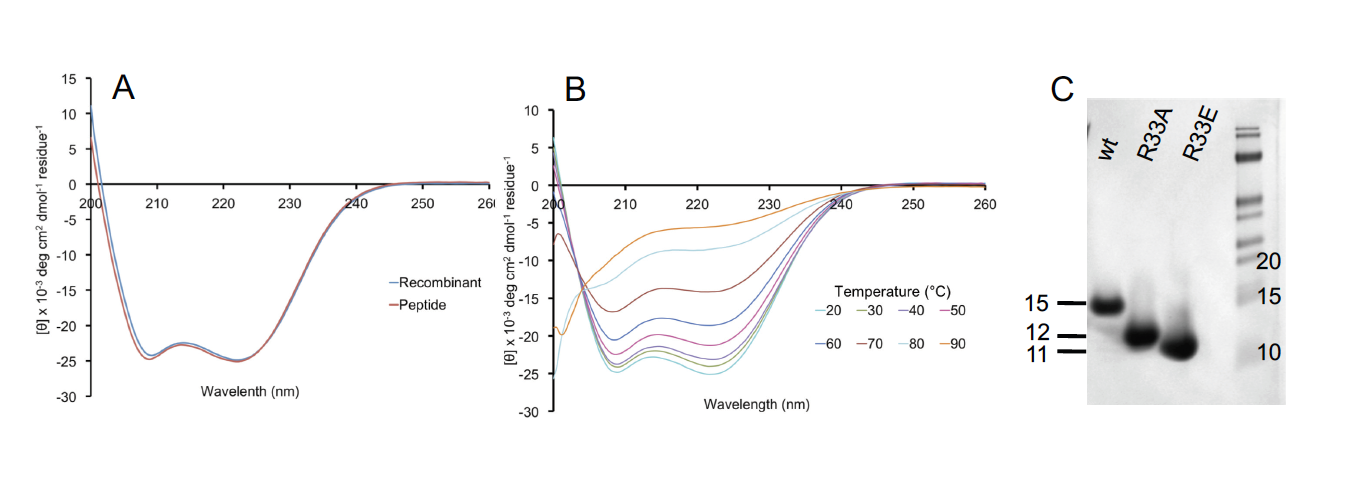


**Figure S1. Recombinant FapF_3-40_ displays same biophysical properties as synthetic peptide.** A) CD curves of the FapF_CC_ peptide and recombinant form, with [θ]_222/208_ ratios of 1.03 and 1.05, respectively B) CD spectra of the FapF_CC_ peptide collected as a function of temperature. C) Native gel electrophoresis of the wild type (wt), R33A and R33E mutants. Approximate molecular weights are shown. SEC-MALS analysis gave the following molecular weights: wt (15.3 ± 5 %); R33A (12.2 ± 4%); R33E (11.0 ± 4 %).


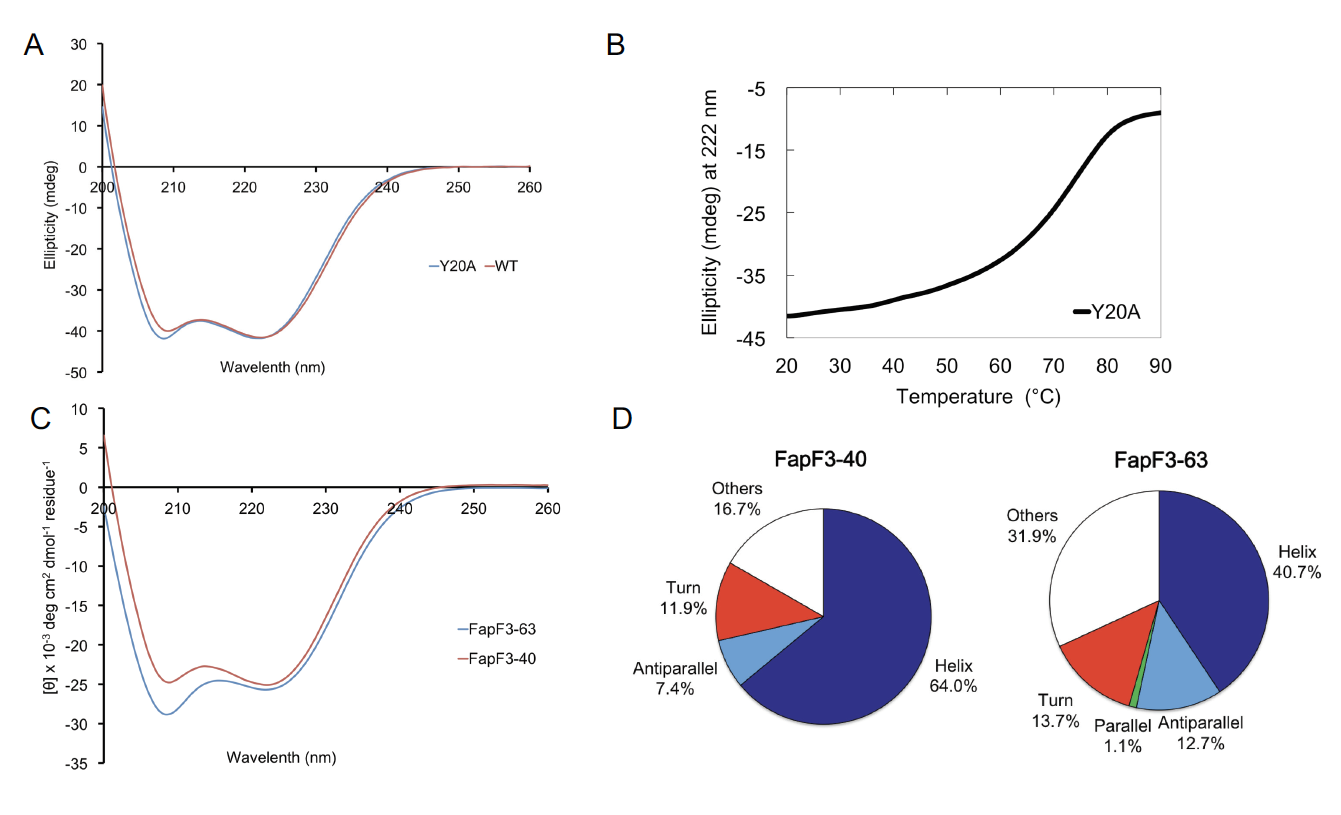
**Figure S2. Biophysical properties of Y20A and an extended FapF_CC_ construct.** A) CD curves show that Y20A does not alter FapF_CC_ secondary structure. B) the CD melting curve profile for Y20A show a similar structural stability to WT (Y20A T_m_ = 73.5 °C). C) CD curves of the FapF_3-63_ and FapF_3-40_. D) Secondary structure composition for FapF_3-40_ and FapF_3-63_ as estimated by the BeStSel method. The extended FapF_CC_ construct, FapF_3-63,_ is consistent with the addition of an unstructured region.

**Figure S3**


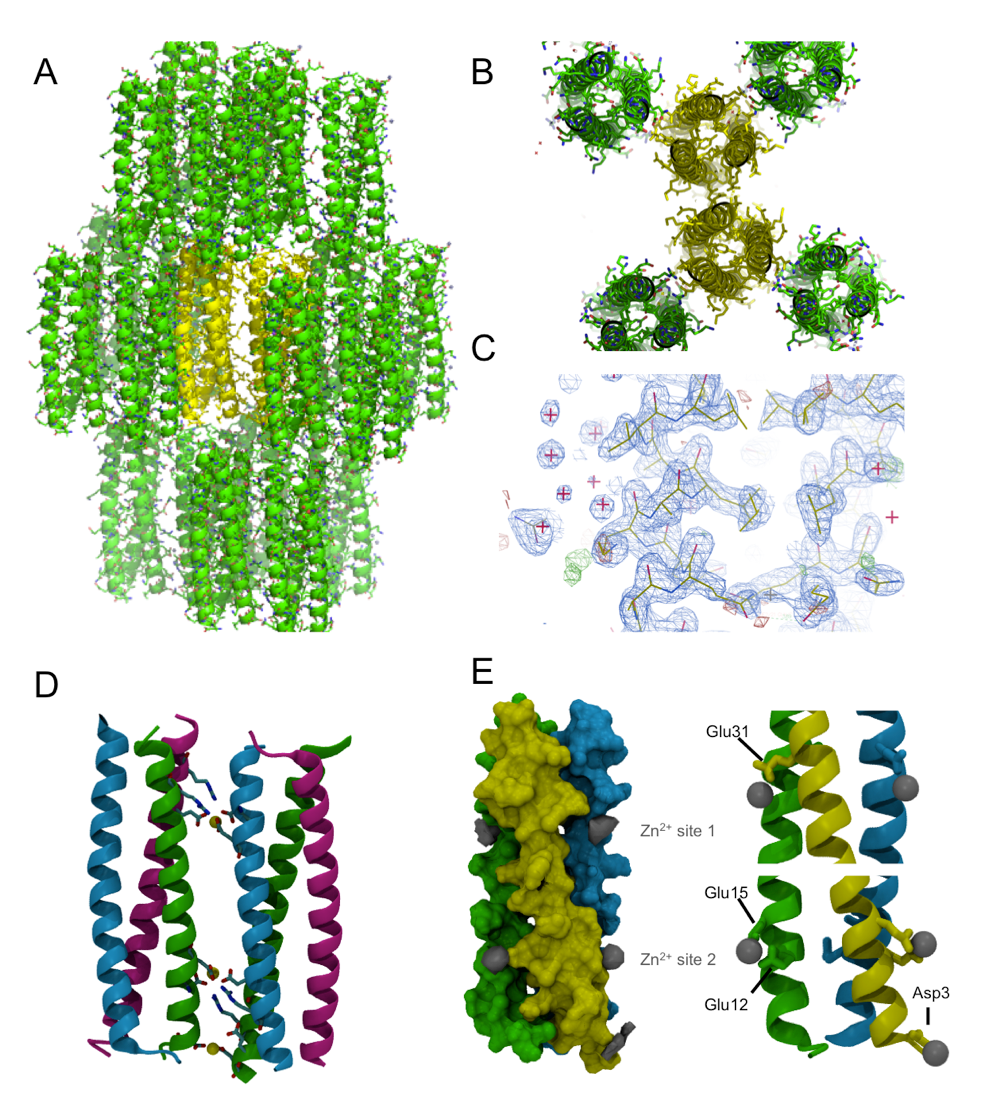


**Figure S3. Packing between trimers in the crystalline state.** A, B) The trimers pack together in an antiparallel fashion with two trimers in the asymmetric unit. C) An example of the chain density map is shown. D) The interaction between trimers is mediated by the presence of Zn^2+^ ions which were required for crystallisation to occur. 3 Zn binding sites between the dimer found in the asymmetric unit are shown with residues within 1 nm shown in stick representation. E) In molecular simulations of a single trimer in solution the Zn^2+^ remained bound throughout the course of each 100 ns simulation. The averaged position of Zn^2+^ over 3 independent 100 ns simulations is shown as a grey surface calculated using the Volmap plugin. A representative snapshot from the end of a single simulation indicating the position of Zn^2+^ (corresponding to Run1 in Figure S3) is shown.

**Figure S3**


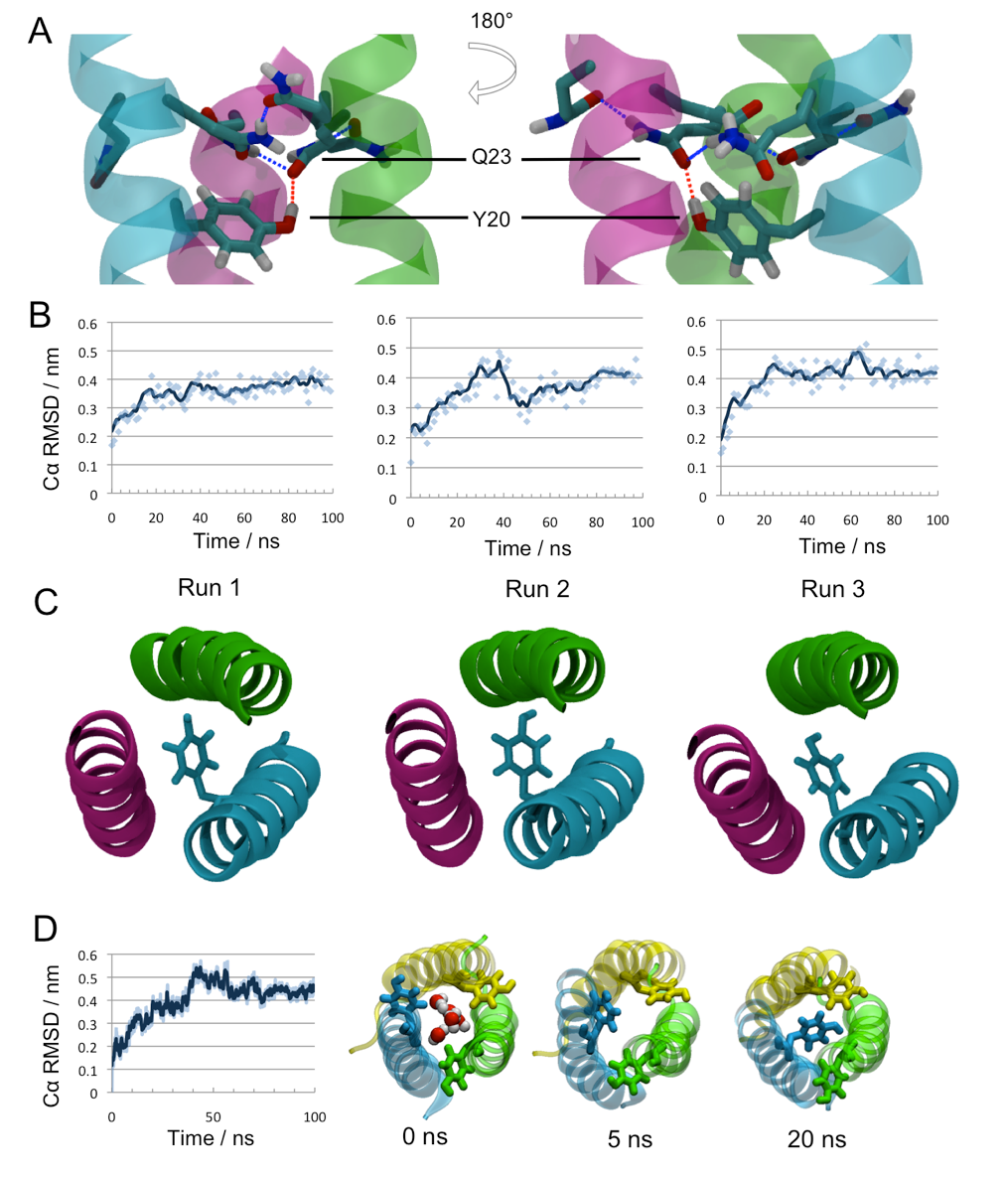


**Figure S4. A single Tyr20 adopts a buried rather than interfacial orientation.** A) Buried Tyr20 is able to hydrogen bond with a neighbouring chain Gln23. B) Cα RMSD plots for simulations of the trimer in 0.15 M NaCl solution. Data points shown as light blue markers, running average over 5 data points shown as a dark blue trace. C) Tyr20 remains buried for the duration of 3 independent 100 ns simulations as indicated by snapshots shown at 100 ns for each simulation. D). Control simulation in which all 3 Tyr20 were modelled in a symmetric interfacial orientation led to a rearrangement of a single sidechain within 10s of ns. The reverse transition was not observed in any simulation of the coiled coil.

**Figure S4**


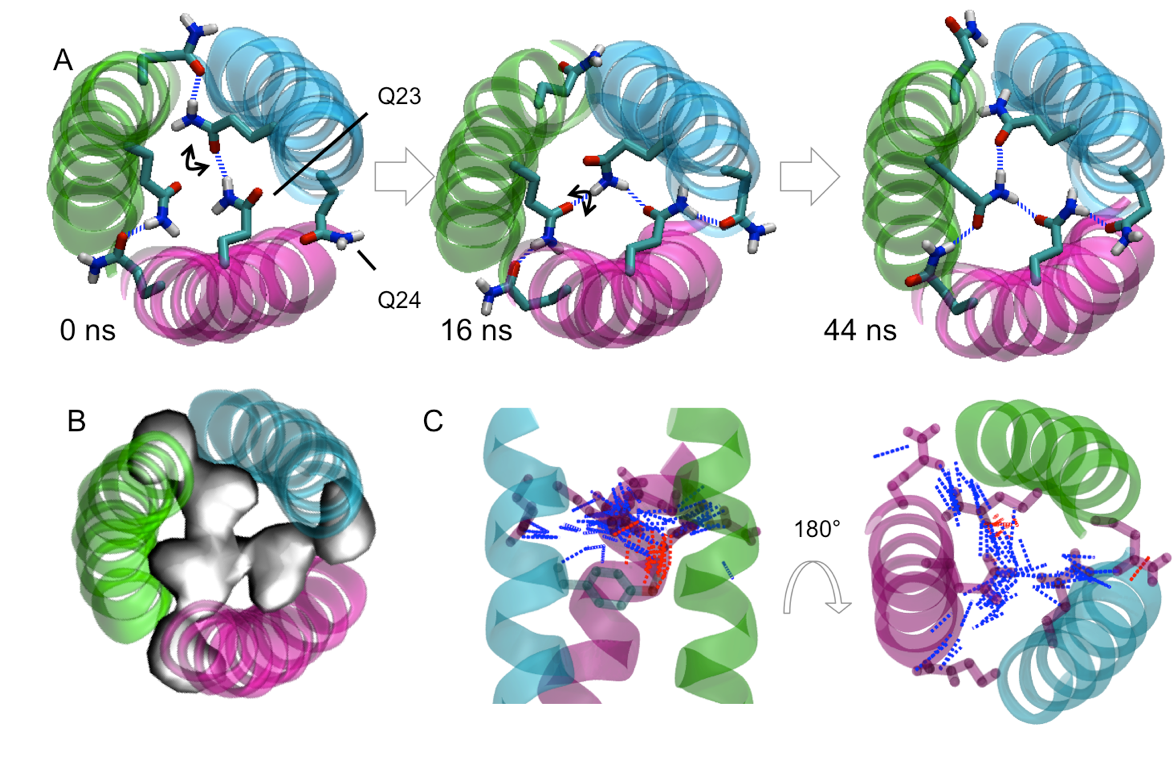


**Figure S5. Dynamic hydrogen bonded network of glutamine residues**. A) Molecular simulations of the coiled-coil in solution captured a range of Gln23-24 conformations, with sidechain switching, highlighted by an arrow, occurring on the ns timescale. Hydrogen bonds between Gln23-24 are shown as dashed lines. Snapshots from simulation Run 1 are shown. B). Averaged positions of Gln23 Gln24 sidechains over three independent trajectories shown as a surface calculated using the Volmap tool. C) Hydrogen bonds between the Gln23, Gln24 and the buried Tyr20 are shown at 5 ns intervals from a single trajectory. The buried Tyr20 sidechain forms part of this extended hydrogen bond network.

**Figure S5**

**
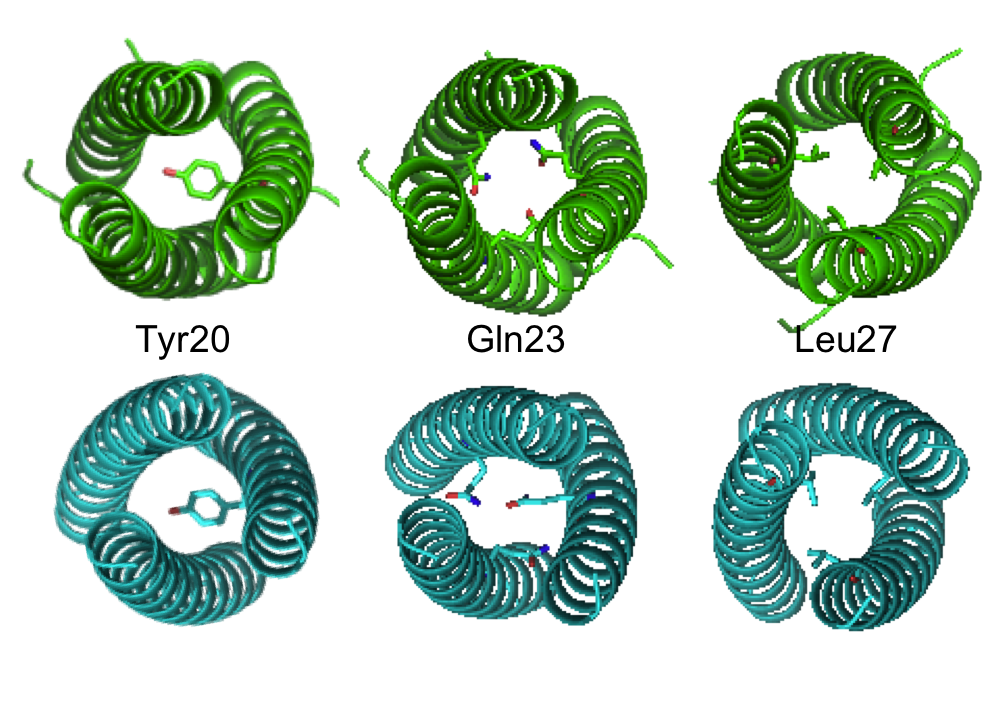
**

**Figure S6. Comparison of previously published CCBuilder v1.0 model of FapF vs the subsequent X-ray structure.** Notably a single Tyr20 was buried in the CCBuilder model.
